# Topological structure analysis of chromatin interaction networks

**DOI:** 10.1186/s12859-019-3237-z

**Published:** 2019-12-27

**Authors:** Juris Viksna, Gatis Melkus, Edgars Celms, Kārlis Čerāns, Karlis Freivalds, Paulis Kikusts, Lelde Lace, Mārtiņš Opmanis, Darta Rituma, Peteris Rucevskis

**Affiliations:** 1 Institute of Mathematics and Computer Science, University of Latvia, Rainis boulevard 29, Riga, LV-1459 Latvia; 2 Faculty of Computing, University of Latvia, Rainis boulevard 19, Riga, LV-1586 Latvia

**Keywords:** Chromatin interaction networks, Graph topology, Cell type specificity, Functionally related modules

## Abstract

**Background:**

Current Hi-C technologies for chromosome conformation capture allow to understand a broad spectrum of functional interactions between genome elements. Although significant progress has been made into analysis of Hi-C data to identify biologically significant features, many questions still remain open, in particular regarding potential biological significance of various topological features that are characteristic for chromatin interaction networks.

**Results:**

It has been previously observed that promoter capture Hi-C (PCHi-C) interaction networks tend to separate easily into well-defined connected components that can be related to certain biological functionality, however, such evidence was based on manual analysis and was limited. Here we present a novel method for analysis of chromatin interaction networks aimed towards identifying characteristic topological features of interaction graphs and confirming their potential significance in chromatin architecture. Our method automatically identifies all connected components with an assigned significance score above a given threshold. These components can be subjected afterwards to different assessment methods for their biological role and/or significance. The method was applied to the largest PCHi-C data set available to date that contains interactions for 17 haematopoietic cell types. The results demonstrate strong evidence of well-pronounced component structure of chromatin interaction networks and provide some characterisation of this component structure. We also performed an indicative assessment of potential biological significance of identified network components with the results confirming that the network components can be related to specific biological functionality.

**Conclusions:**

The obtained results show that the topological structure of chromatin interaction networks can be well described in terms of isolated connected components of the network and that formation of these components can be often explained by biological features of functionally related gene modules. The presented method allows automatic identification of all such components and evaluation of their significance in PCHi-C dataset for 17 haematopoietic cell types. The method can be adapted for exploration of other chromatin interaction data sets that include information about sufficiently large number of different cell types, and, in principle, also for analysis of other kinds of cell type-specific networks.

## Background

The basic functionality of a genome is regulated on many levels, especially regarding how and when individual genes are transcribed and subsequently translated into proteins. The timely, specific and accurate regulation of these processes is the key to the proper functioning of an organism. Most of an organism’s regulatory processes originate in its own genome, two primary categories of these processes can be delineated based on whether they are interactions primarily between regions of DNA (cis-regulation) or between DNA and another molecule such as a regulatory protein (trans-regulation). Both of these forms of interaction are deeply involved in the essential mechanism underlying the action of transcription factors: the promoter-enhancer interaction (PEI). The PEI links together a promoter, the region encompassing the vicinity of a transcription start site of a gene and the target of the transcription factor binding, and an enhancer, a distantly positioned genomic region that acts as a facilitator of the transcription. When active, a promoter and its corresponding set of enhancers are connected with the help of activator proteins and other regulatory factors, and form a small, distinctive chromatin loop [1–3].

Promoter-enhancer interactions are central to the current understanding of transcriptional regulation, and studying them requires methods which are able to capture the location, prevalence and changes of the chromatin looping. The method most directly targeted at capturing these chromatin features thus far has been chromatin conformation capture or 3C, originated almost two decades ago [4] and subsequently advanced with the advent of next-generation sequencing to create Hi-C [5]. Hi-C is theoretically capable of capturing all chromatin interactions between two genomic sites, however, in its classic form it is generally insensitive to PEIs due to rarely providing reliable data at high enough resolutions to capture small-scale chromatin looping [6–8]. Several approaches exist to improve this resolution, including much greater sequencing depths [9] as well as superior restriction enzymes and other technical improvements [7]. One particular method, capture Hi-C (CHi-C), adds a sequence capture step that pares down the range of interactions to a smaller subset associated with defined genomic regions (’baits’) [10, 11]. This allows for easier capture of a particular kind of interaction and has been successfully applied to study PEIs specifically in a variant of the method called promoter capture Hi-C, or PCHi-C [11, 12]. The single largest PCHi-C study to date was conducted by Javierre and colleagues [12] on a variety of human cells belonging to the haematopoietic lineage. This study found that each particular lineage of cells had noticeable differences in the distribution of its PEIs and that many of these interactions appeared to match up with putative genetic variations linked to disease and altered gene expression. Due to its excellent coverage of a range of closely related non-cancerous cell lineages the study provides an attractive target for further analysis, particularly for using computational methods to find patterns in chromatin dynamics.

Analysing Hi-C data is often non-trivial. Many techniques can be employed to improve the fidelity of a given dataset, from more effective experimental and data processing pipelines to post-processing methods for finding particular features within a dataset [13–15]. Fewer analytical methods, however, focus on the dynamics of the chromatin architecture and its specificity [16]. Clustering techniques in particular may be useful in detecting functional modules of genes in Hi-C data. This can be observed in approaches such as Arboretum-Hi-C, which use spectral clustering to identify groups of interactions conserved between several Hi-C datasets [17], or GraphTeams, where a *δ*-teams model is used to locate gene clusters in Hi-C data [18]. Similar techniques may be useful in detecting features in chromatin interaction landscapes that map to broader mechanisms of regulatory interaction such as transcription factories [19] or activity hubs [20]. This makes clustering a potentially valuable technique for interpreting PCHi-C datasets, where functional modules made up of PEIs rarely caught in lower-resolution Hi-C data might be isolated. To our knowledge, however, the employment of topology-based clustering approach in the analysis of capture Hi-C data still has been quite limited – the approach has been used in our own previous related work [21] and for (ChIA-PET network analysis) in [22].

From the topological features of PCHi-C interactions analysed in [21] the most notable was the observation that PCHi-C interaction networks tend to separate easily into comparatively small and well-defined connected components when we restrict the analysis to the networks in which all interactions are required to be present in several (two or more) different cell types. It was also observed that connected components of interaction networks have a tendency to remain largely unchanged when the presence of the same interactions is additionally required for a number of other (component-specific) cell types and largely (or completely) disappear when the presence of the same interactions is additionally required for several other (component-specific) cell types. Such a feature provides an additional indication that PCHi-C interaction network components are likely related to some biological functionality and for several components manually selected for further biological validation their relation to functionally related gene modules has been shown [21]. Still, in [21] the network components were obtained by a manual ad-hoc procedure (by choosing a certain subset of cell types and visually exploring how the networks change when other cell types are either added or removed), leaving open the question how pronounced such component structure is, and the problem of automated identification of ’potentially interesting’ components remaining a challenge.

This behaviour was observed for the network describing PCHi-C interactions for 17 different haematopoietic cell types. It would have been very interesting to check whether the same type of behaviour is characteristic for PCHi-C interaction networks for different sets of cell types, however, to our knowledge no other data set suitable for such a purpose has yet become available. Due to this, we are focusing our analysis on the available PCHi-C interaction networks for 17 haematopoietic cell types.

The identification of network components is related to the problem of comparison of one or more networks, which has been widely studied from various perspectives, and with particularly strong focus on biomolecular networks. For the latter, probably one of the most successful approaches has been based on graphlet sampling ([23–25]). However, in the our case comparison of two networks is not an issue (since the networks are labelled this can be done in linear time), the task is to identify potentially significant or interesting components that discriminate between 2^*k*^ networks assigned to each subset of *k* objects (cell types). The problem is quite specific and, as far as we know, has not been studied before. In slightly generalised terms it can be described as follows.

**Fig. 1:**
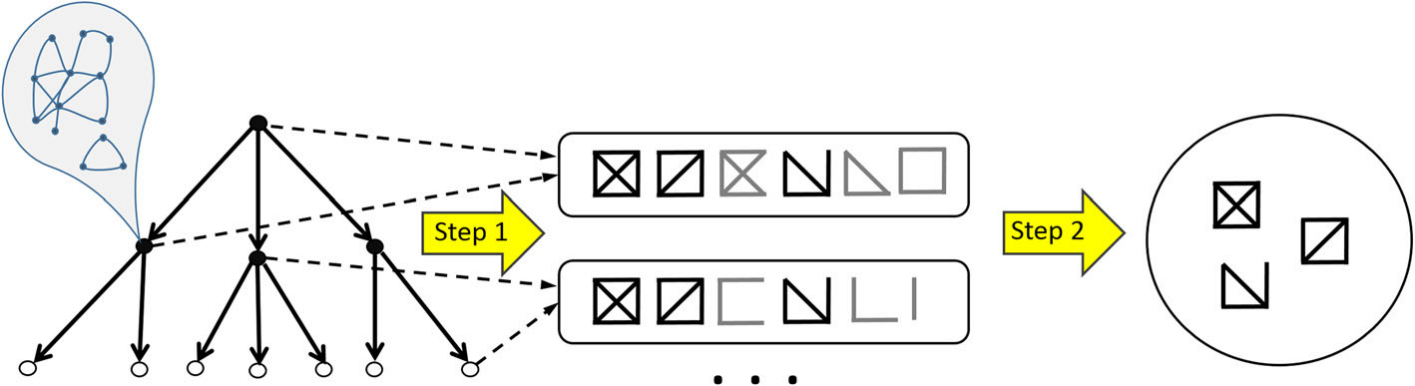
Overview of method for identifying network components. The initial data are represented by a rooted tree with graphs (networks) assigned to its nodes in such a way that graphs assigned to child nodes are (not necessarily proper) subgraphs of the graph assigned to their parent node. The problem is to find a set of ’characteristic’ components (subgraphs) that best differentiate between all the pairs of graphs assigned to the nodes. Although this can be done by a straightforward two-step approach: 1) finding such characteristic set for all pairs of nodes, and 2) combining these sets into a single set that does not contain identical or very similar components, the proposed method does this by a one-pass traversal of the tree significantly reducing time and space requirements

The initial data are represented by a rooted tree with graphs (networks) assigned to its nodes in such a way that graphs assigned to child nodes are (not necessarily proper) subgraphs of the graph assigned to their parent node. (In our particular case this rooted tree is a binomial tree with its vertices corresponding to all the possible 2^*k*^ subsets of set consisting of *k* cell types.) The objective is to find a set of ’characteristic’ components (subgraphs) that best differentiate between all the pairs of graphs assigned to the nodes. In principle this can be done by a straightforward two-step approach: 1) finding such characteristic set for all the pairs of nodes, and 2) combining these sets into a single set that does not contain identical or very similar components. However, such approach requires high time and space complexity; in particular, the complexity of the second step depends on both the number of nodes and sizes of networks, and for whole-genome chromatin interaction networks poses a challenge even for small number (e.g. 17, as in our case) of cell types. The alternative method that we propose does this by a one-pass traversal of the tree and has significantly lower time and space requirements. The overview of the problem and the method is illustrated in Fig. 1.

The proposed approach is particularly suited for identification of connected components, but in principle could be adapted for identification of certain other characteristic topological features of the networks. It is also worth to note that whilst the networks are directed (with edges from ’baits’ to ’other ends’), the edge direction is currently not taken into account in exploring network component structure. There are two (related) main reasons for this: 1) there are different alternatives how to define directed components (the use of strongly connected components is the most obvious one, and we have also partially explored this option; however, it does not seem too promising, one of the problems being that they cover only a small part of the networks – due to the fact that all the nodes must be both ’baits’ and ’other ends’); 2) although there is limited evidence of the importance of edge directionality (e.g. statistical significance of the presence of cycles of length 2 and of bi-connectivity has been observed in [21]), the role of edge directionality is still insufficiently understood to take it into account when analysing the network component structure.

The main contributions of this paper are: 1) demonstration of strong evidence of well-pronounced component structure of chromatin interaction networks and the fact that many of these components tend to be largely preserved in some of cell types and largely absent in some others; 2) a method for automatic detection of all the possible candidates for potentially biologically interesting (as measured by a scoring function assessing their significance) of such network components; 3) characterisation of the component structure of PCHi-C interaction networks gained from the network analysis using this method; 4) assessment of the potential biological significance of identified network components by a ’high-throughput’ approach, whose results confirm that the components tend to be related to specific biological mechanisms/functionality.

However, whilst the limited assessment of the biological significance described here shows statistically significant relation of components to gene regulation patterns, it is very likely that the component structure is the result of several different interrelated biological processes and in this paper we do not attempt to assign to them a conclusive well-defined biological explanation. What we think is significant, is the fact that the component structure of chromatin interaction networks is sufficiently well manifested to be taken into consideration when analysing these networks. Here we propose a method that can be used for efficient automatic discovery of such components, which afterwards can be subjected to different assessment methods for their biological role and/or significance.

## Methods

### Data sets used for network analysis and result validation

**Fig. 2:**
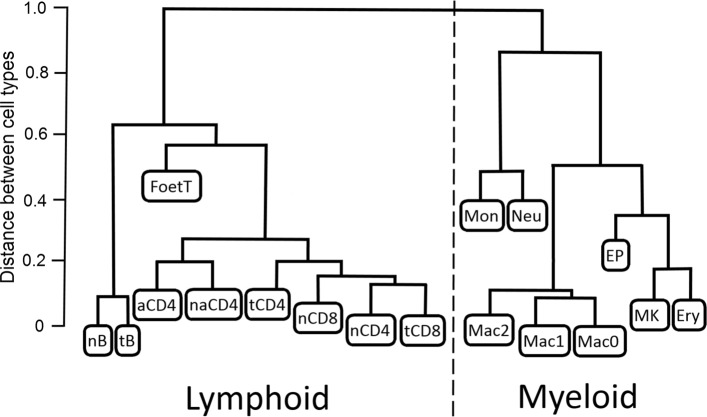
Haematopoietic tree for 17 cell types. 17 haematopoietic cell types in PCHi-C data set and their hierarchical clustering based on distances from [12]. For convenience the distances are re-scaled to produce tree of hight 1

For analysis of PCHi-C interaction network topology we use a data set of long-range interactions between promoters and other regulatory elements that was generated by The Babraham Institute and University of Cambridge [12]. This data set is still largely unique because it contains genome-wide data covering a representative subset of the entire haematopoietic lineage collected using a unified protocol. The data was obtained by promoter capture Hi-C (PCHi-C) in 17 human primary haematopoietic cell types (shown in Fig. 2), and from 31253 identified promoter interaction regions across all chromosomes, a subset of high-confidence PCHi-C interactions have been selected using CHiCAGO pipeline [26]. These data are available from the Open Science Framework website [12].

For the assessment of the potential biological significance of the found network components, several approaches were considered based on possibilities to perform assessments in a ’high-throughput’ manner. The options, however, are limited by the availability of datasets covering the particular 17 haematopoietic cell types. The most straightforward approaches are looking for the similarity of regulation or expression patterns of genes from the same component. Regarding the gene expression, there are few gene data sets that could be used (e.g. BLUEPRINT RNA-seq data set EGAS00001000327), however, the coverage of the gene set for which there are PCHi-C interaction data is rather poor. More accurate data related to the gene expression could be obtained from FANTOM5 promoter level expression atlas [27]. This data set contains transcription start site activity data obtained by CAGE [28] protocol and the available data cover genome-wide information about 14 human haematopoietic cell types, 11 of these overlap with cell types for which PCHi-C interaction data are available. Thus, this data set might seem to be very appropriate for biological validation, unfortunately the coverage again is quite poor – the expression data are available for approximately 18% of vertices (corresponding to ’bait’ ends) of PCHi-C interaction graphs constructed from [12] data.

Regarding the gene regulation patterns, a well-suited data set is chromatin annotations generated through ChromHMM based on ChIP data from BLUEPRINT [29]. The dataset includes information on transcription factor binding sites, CTCF binding sites, DNase-sensitive sites, transcription start sites, proximal and distal cis-regulatory regions, as well as the activity modes of transcription start sites and cis-regulatory regions, as observed from the aforementioned annotations. The same annotations were previously used in the original publication [12] to validate the interactions between promoters and regulatory elements, but their analysis did not focus on the discovery of potential hubs of the activity or coordinated repression. The annotations are available for 9 of 17 cell types for which there are PCHi-C data and vertex coverage is also quite good. For this reason we have chosen to use it here for assessment of biological significance of the identified topological features (connected network components with significance scores above a certain threshold). The data are available from Open Science Framework website [12].

### Graph representation of PCHi-C interaction networks

Technically the network of PCHi-C interactions is described as a digraph (directed graph) *G* with a set of vertices *V*=*V*(*G*) consisting of promoters (’baits’) and detected interaction regions (’other ends’). (A vertex can also be both: a ’bait’ and an ’other end’.) For convenience, however, such vertex set *V* we represent simply as a set of integers {1,…,*n*}, where *n*=|*V*|.

The set of edges *E*=*E*(*G*) corresponds to detected interactions and edges are directed from ’baits’ to ’other ends’ (it is possible that for some vertices *v*_1_,*v*_2_∈*V* both edges (*v*_1_,*v*_2_)∈*E* and (*v*_2_,*v*_1_)∈*E*). When constructed from Hi-C data, the set of edges *E* usually will depend on the selected interaction score threshold (only interactions with scores not below the threshold will be chosen for edges). For dataset analysed here we use the same threshold that was proposed in [12] – i.e. only interactions with score 5 or above are selected for edges.

By $\mathcal {T}$ we denote the set of all the available cell types. For convenience we also assume that these cell types are indexed, i.e. $\mathcal {T}=\{t_{1},\ldots,t_{k}\}$, where *k* is the number of available cell types. (For dataset in our study we have *k*=17.) For $t\in \mathcal {T}$ its index is denoted by *idx*(*t*) for set $T\subseteq \mathcal {T}$ we define *idx*(*T*)=*max*{*idx*(*t*)∣*t*∈*T*} – i.e. the maximal index of *T* elements.

For each edge *e*∈*E* there is assigned a non-empty set of labels $T(e)\subseteq \mathcal {T}$. These labels correspond to cell types for which the scores for the interaction reached at least the threshold level. For $T\subseteq \mathcal {T}$ by *G*(*T*) we denote a subgraph of *G* with vertex set *V* and edge set {*e*∈*E*∣*T*⊆*T*(*e*)}.

A *connected component* (or CC) of a digraph *H* here we simply will define as a connected component of the corresponding undirected graph $\hat {H}$, obtained from *H* by ignoring edge directions (i.e. we will not require strong connectivity of components). The fact that *C* is a connected component of digraph *H* we denote by *C*≤_*cc*_*H*. For $T\subseteq \mathcal {T}$ and *C*≤_*cc*_*G*(*T*) by *G*(*C,T*) we denote a subgraph of *G*(*T*) with set of vertices restricted to *V*(*C*) – this will be useful to discuss changes of component *C* when *T* defining it is replaced by another set *T*^′^.

### Algorithm for network analysis

Our aim is to find the set of all connected components $\{C \leq _{cc} G(T) \mid T\subseteq \mathcal {T}\}$ that are ’medium-sized’ (with number of vertices *n* between some thresholds *n*_*min*_ and *n*_*max*_) and have ’sufficiently interesting properties’ to merit further exploration of their potential biological significance (this is assessed by function SIGNIFICANCESCORE, a reasonably good candidate for which we are proposing below, but the function likely could be further elaborated and/or modified to improve its biological accuracy or to adapt it for assessment of different kinds of biological features).

For identification of all such connected components, we propose the algorithm FINDNETWORKCOMPONENTS (Algorithm 1) described below. The algorithm is based on BFS (Breadth First Search) of binomial tree defined by subsets of $\mathcal {T}$ (with *k*+1 levels *i*=0…*k*) and all the possible *i* element subsets of $\mathcal {T}$ at level *i*).

**Fig. 3:**
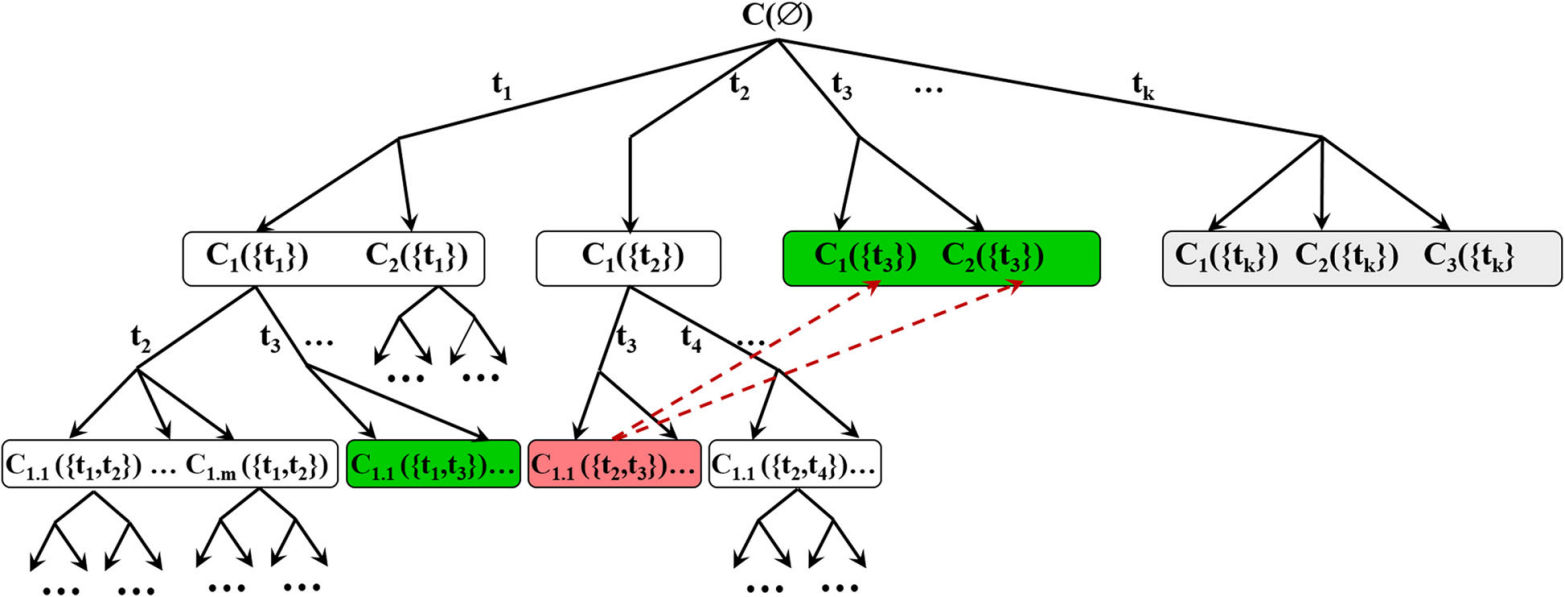
Search tree of FINDNETWORKCOMPONENTS algorithm. Schematic representation of part (limited to a single CC at the root) of binomial search tree constructed and traversed by FINDNETWORKCOMPONENTS algorithm. The binomial tree corresponding to all subsets of $\mathcal {T}$ is additionally branched due to the possibility of CCs to split into several at the next depth level. The search tree is pruned: 1) below components with less than *n*_*min*_ vertices (these are simply not added to the tree); 2) below components reaching significance score threshold (components *C*_1,1_({*t*_1_,*t*_3_}),*C*_2_({*t*_3_}),*C*_2_({*t*_3_})); 3) components *C*(*T*) that are subgraphs of already found components *C*^′^(*T*^′^) with *T*^′^⊆*T* (component *C*_1,1_({*t*_2_,*t*_3_})). Here for brevity *C*(*T*) is used to denote *G*(*C,T*)


At each depth level *i*=0...*k* queue *Q*_*i*_ initially contains all the connected components *C*≤_*cc*_*G*(*T*) for all $T\subseteq \mathcal {T}$ with |*T*|=*i* satisfying certain pruning criteria. *Q*_*i*_ also contains components *C*≤_*cc*_*G*(*T*^′^) with |*T*^′^|<*i* further analysis of which have been pruned earlier and which are marked as inactive. During the processing of *Q*_*i*_ components with significance score reaching threshold *s* or above are printed and the queue *Q*_*i*+1_ for the next level of the binomial tree is constructed. The construction of *Q*_*i*+1_ is omitted for the last depth level *i*=*k*. The algorithm prints a list of all components, together with subsets of cell types $T\subseteq \mathcal {T}$ defining them and their significance scores. Schematically the search tree constructed by the algorithm is shown in Fig. 3.

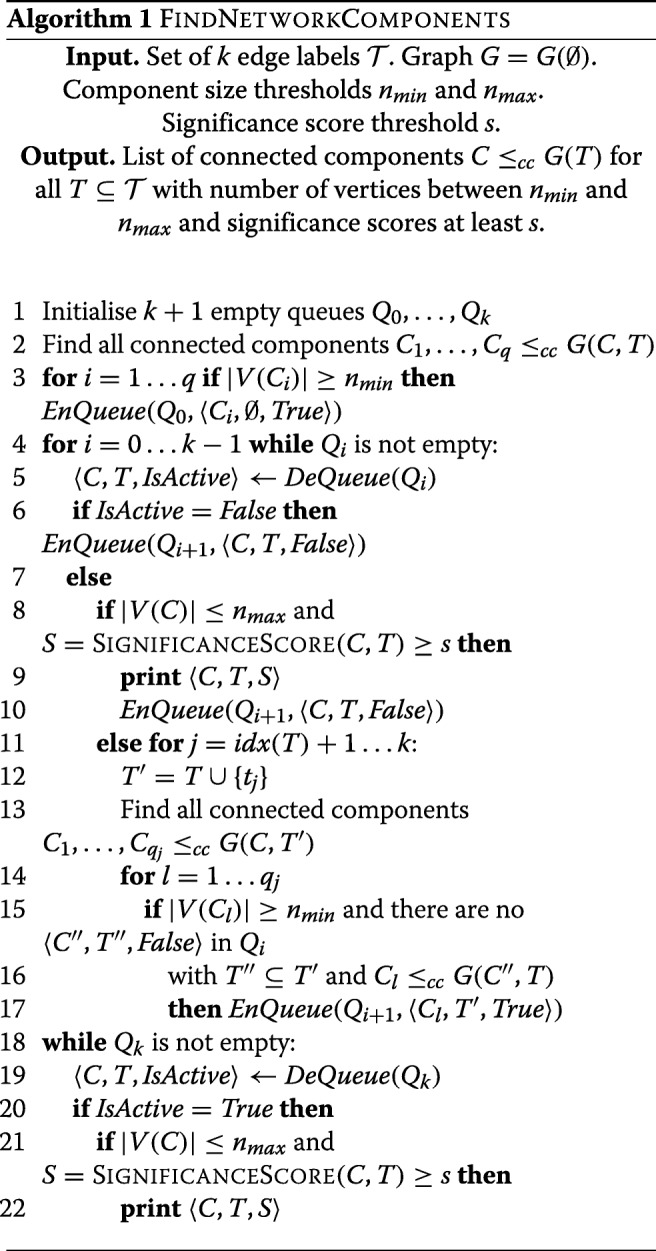


The algorithm is called on initial graph *G*=*G*(*∅*) that is assumed to be based on interactions for all the chromosomes, however, since there are very few interactions between different chromosomes, technically it is more convenient to build and analyse *G* for each of 23 chromosomes separately. For our dataset, there seems to be a good motivation to chose *n*_*min*_=10, the choice of *n*_*max*_ is more arbitrary, we used *n*_*max*_=100 to limit components to a manageable size for further analysis. The value of *s* depends on the properties of function SIGNIFICANCESCORE; in our case by its design it was appropriate to choose *s*=0.

The purpose of SIGNIFICANCESCORE function is to guess/predict how ’interesting’ from the biological perspective the detected CCs could be. Since we are mostly interested in the variability of CCs for different cell types, it is natural to ask that for a *C*≤_*cc*_*G*(*T*) with *n*=|*V*(*C*)| vertices and *m*=|*E*(*C*)| edges there is a cell type $t^{\prime }\in \mathcal {T}-T$, such that *G*^′^=*G*(*C,T*∪{*t*^′^}) preserves most of *C* edges, and also a cell type $t^{\prime \prime }\in \mathcal {T}-T$, such that *G*^′′^=*G*(*C,T*∪{*t*^′′^}) preserves only few of *C* edges. It is also natural to score higher CCs that have more options for choice of such types *t*^′^ and *t*^′′^. Often used and well suited choice for a scoring function with such properties is (for easier readability denoting the number of edges |*E*(*H*)| of a graph *H* by *e*(*H*)):

$$\begin{array}{*{20}l} & \textsc{SignificanceScore}(C,T) = & \\ &\quad (|\{t^{\prime}\in \mathcal{T}-T \mid e(G^{\prime})\geq a\cdot e(C)\}| \cdot |\{t^{\prime\prime}\in \mathcal{T} \\ &\quad -T \mid e(G^{\prime\prime}) \leq b\cdot e(C)\}|)^{1/2} & \\ \end{array} $$

The constants *a* and *b* should satisfy 0≤*b*<*a*≤1 and empirically were chosen as *a*=0.75 and *b*=0.25, (for the data set used it was also observed that slight variations of them do not have a significant impact on the results – see Table 1). The square root taken from the product of cardinalities of two sets does not affect the ordering of significance scores, but provides more linear distribution of their values.

**Table 1:** Dependence of the number of discovered connected components from constants *a* and *b* in SIGNIFICANCESCORE evaluation function

	*a*=0.70	*a*=0.75	*a*=0.80
*b*=0.20	3349	3454	3773
*b*=0.25	3821	3966	4325
*b*=0.30	4318	4490	4871

These data are for chromosome 1

**Algorithm complexity**

The algorithm involves traversing 2^*k*^ vertices corresponding to all the possible subsets $T\subseteq \mathcal {T}$ thus its time complexity unavoidably is exponential in *k*. Most of the tasks involving the processing of graphs (splitting into CCs, computing *C*(*T,E*)) can be done in time *O*(*m*) (where *n* and *m* correspondingly are numbers of graph vertices and edges). Checking whether a constructed CCs *C* should be pruned (lines 14,15,16 of the code) can be done in time *O*(2^*k*^|*V*(*C*)|).

This gives the overall algorithm time complexity *O*(2^2*k*^*n*+2^*k*^*m*). The main space requirements are for storage of two queues containing information for up to $\binom {k}{\lfloor k/2 \rfloor } n$ graph vertices in each, thus algorithm space complexity is *O*(2^*k*^*n*).

These bounds, however, are not very informative for comparatively low values of *n*, *m* and *k* for our particular PCHi-C data set, since the actual complexity depends on the number of CCs constructed by the algorithm and the number of pruned search tree branches as well as levels at which the pruning occurs. For this dataset we have *k*=17 and, depending on the chromosome, *n* varies between 2000 and 18000 vertices and *m* between 3000 and 44000 edges. For these values the number of CCs is notably lower and the extent of pruning notably higher than the indicative asymptotic bounds.

In practice the current C++ implementation of the algorithm (on a single core of Xeon E5-1620 v3 3.50GHz processor) for processing of single chromosome data required up to 7 minutes of running time and up to 300 MB of RAM. Thus, the algorithm is quite practical for analysis of genome-wide Hi-C interaction data for *k*=17 cell types, however, exponentiality in *k* also means 17 cell types could be already quite close to the upper limit to which the algorithm can be directly applied.

### Relation with previous work and alternative approaches

The fact that PCHi-C interaction network *G*(*T*) separates easily into comparatively small (with sizes up to few hundred vertices) connected components, when set of cell types *T* contains two or more (sufficiently distinct) haematopoietic cell types was observed by the authors in [21] by the exploration of visualised interaction networks. It was also observed that connected components have a tendency to remain largely unchanged when shared by a number of additional different (component-specific) cell types and largely (or completely) disappear when shared by some other (component specific) cell types. A component structure of *G*(*T*) for several particular choices of the set of cell types *T* was explored in more detail, and few components were chosen for analysis of their biological significance. It was shown that these selected components form functionally related gene modules.

The exact component structure of *G*(*T*), however, depends on the choice of *T*, and one of the main contributions of this work is the algorithm for automatic detection of all connected components *C*≤_*cc*_*G*(*T*), which are assessed as potentially biologically significant, for all the possible choices of $T\subseteq \mathcal {T}$. The algorithm also prunes the list of components found by not searching for any sub-components *C*^′^≤_*cc*_*G*(*C,T*^′^), if component *C*≤_*cc*_*G*(*T*) has already been output as significant for some *T*⊆*T*^′^. The pruning, however, still does not exclude only partially overlapping components, or components of *G*(*T*) and *G*(*T*^′^) for only partially overlapping *T* and *T*^′^.

The direct ’automatisation’ of manual choosing of sets of cell types *T* that produces *G*(*T*) with well-defined component structure (which was attempted in [21]) could be achieved by a similar but simpler algorithm based on BFS of binomial tree defined by subsets of $\mathcal {T}$ and separate analysis of the component structure of whole graphs *G*(*T*) associated with the corresponding vertices of the binomial tree. However, it is not difficult to show that the proposed analysis at the level of individual components of *G*(*T*) instead of whole graphs *G*(*T*) still outputs all the components that will be assessed as significant by SIGNIFICANCESCORE scoring function. An additional notable benefit is a significant reduction of the number of components output by the search procedure due to omitting of components that are identified as subgraphs of others.

### Assessment of the biological significance

The strongly pronounced tendency of chromatin interaction networks to split into well defined medium sized connected components is clearly not a feature shared by random graphs and very likely should be explained by some biological reasons. Whilst there is also a possibility that at least partially this topological feature might be the result of limited freedom of possible conformations of chromatin 3D structure, even in this case it should likely have some impact of correlated functionality of genes contained in a particular component.

At the same time, it is very unlikely that this component structure could be attributed to a single and clearly defined biological mechanism, and these components should be regarded as good candidates for modules of genes on which to focus attention for a more thorough exploration of their potential biological significance.

In [21] we have analysed a number of selected components for enrichment with registered transcription factor protein-protein interactions, known transcription factor binding sites, co-expressed transcription factors and binding motifs with the *Enrichr* web tool [30, 31]. It was found that genes contained in specific components tend to be associated with common transcription factors. Such analysis, however, is difficult to perform for a large number (few tens of thousands) of components that are identified automatically.

Here we have used another more ’high-throughput’ approach for assessment of the potential biological significance of network components. It is based on looking for relations of components to activity modes of the involved transcription start sites, proximal and distal cis-regulatory regions using chromatin annotation data generated through ChromHMM based on ChIP data. The data set assigns transcription start sites (TSS) and/or regulatory regions to a number (not all) interactions in PCHi-C network, which are further characterised by 4 different activity modes denoted by 0 (’dead’),1 (’active’), 2 (’poised’),3 (’Polycomb-repressed’). These assignments are specific for each of cell types from $\mathcal {T}$ and the data are provided for 9 different cell types.

To assess whether there is a tendency for network components to be associated with certain activity modes, for each component *C* defined by $T\subset \mathcal {T}$ and for each cell type *t*∈*T*, for which data is available, we construct a 4-tuple 〈*a*_0_,*a*_1_,*a*_2_,*a*_3_〉. *a*_*i*_ value is equal to the number of edges *e*∈*E*(*G*(*C*,{*t*})) to which is assigned activity mode *i*, multiplied by 4-tuple specific normalisation constant *c*, chosen to ensure that *a*_0_+*a*_1_+*a*_2_+*a*_3_=1. Thus, to each component *C* there is assigned a *r*×4 size matrix (where *r* depends on the number of available data entries), with rows consisting of 4-tuples for these *t*∈*T* for which data are available. The largest possible value or *r* is 9, and we consider only components with *r*>1. The level of association of *C* with particular activity mode *i* can then be assessed by comparing the variance of *a*_*i*_ values within matrix assigned to *C* with the overall variance of *a*_*i*_ values and/or with the variance of *a*_*i*_ values for randomised data. Another option is comparing the distribution of average or maximal values of *a*_*i*_ within components with the average or maximal values for randomised data.

## Results

### Topological features of PCHi-C networks

**Fig. 4:**
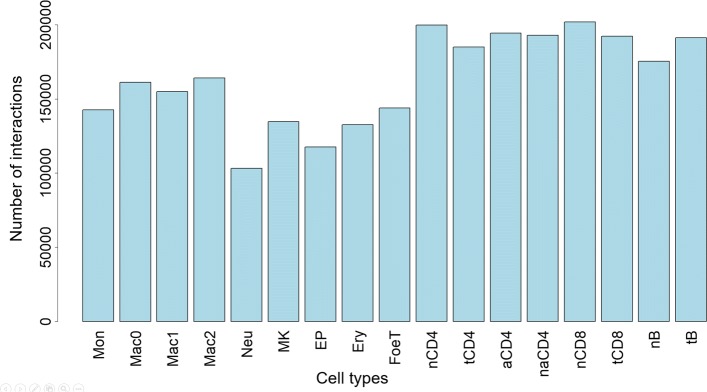
Number of interactions for different cell types. The number of PCHi-C interactions for each of 17 haematopoietic cell types. This is the total number for 23 chromosomes (i.e. all chromosomes, except Y) used for interaction network analysis

Since there are almost no interactions between different chromosomes in the available PCHi-C dataset (from [12]), it was natural to construct and analyse interaction networks separately for each chromosome. The data set also contains very few interactions for Y chromosome, thus only interaction networks for chromosomes 1…22 and chromosome *X* were analysed (for technical convenience the latter here is also called ’chromosome 23’). The number of vertices in the chromosome-specific networks ranges between 2904 (chromosome 22) and 23079 (chromosome 1), the overall number of vertices in 23 interaction networks is 251209 and the overall number of edges is 723165. The number of edges to which each of 17 cell types is assigned varies between 100000 and 200000 (see Fig. 4).

The fact that PCHi-C interaction networks *G*(*T*) separate easily into comparatively small connected components was already observed by the authors in [21]. For *T*=*∅* (i.e. no requirement for edge presence in any particular cell type) the networks, however, consist of one large component containing 40−75*%* of *G*(*T*) vertices, the other CCs are noticeably smaller (except for chromosome 18 for which the two largest components correspondingly contain 1925 and 1178 vertices), and there are very few (2 to 14) CCs with 10 or more vertices (the choice of *n*_*min*_=10 is somewhat arbitrary, but quite well suited to remove ’random noise’). The well-defined structure of medium-sized components start to appear when *T* contains 2 or more not too similar cell types – the sizes of the largest CCs tend to drop below 150 and there are about 50 (on average, the numbers are generally proportional to the size of *G*(*T*)) components with 10 or more vertices per chromosome.

For finding connected components FINDNETWORKCOMPONENTS algorithm was tested with parameters *n*_*min*_=10,*s*=0 (the only natural choice this particular SIGNIFICANCESCORE function) and with different values of *n*_*max*_ and of *a* and *b* in the SIGNIFICANCESCORE function.

The value of *n*_*max*_ should not be too large (to limit components to manageable size for further analysis) but also not too small (there is a quite large number of components with 70−80 vertices and high significance scores). Increasing *n*_*max*_, however, leads to the output of fewer components and *n*_*max*_=100 was selected as a convenient choice from interval where small variations of *n*_*max*_ do not significantly affect the number of components found by the algorithm.

The effect of the choices of *a* and *b* on the number of components found is illustrated in Table 1 and the choice of the retained edge level thresholds *a*=0.75 and *b*=0.25 can be considered as a reasonably good compromise. An interesting seemingly counter-intuitive feature is an increase of the number of components for higher *a* values, which is due to the pruning occurring at lower levels (as a result the algorithm outputs several partially overlapping subgraphs of a larger component that by itself fails the score). However, there is no obvious choice for the ’optimal value’ of *a*.

**Fig. 5:**
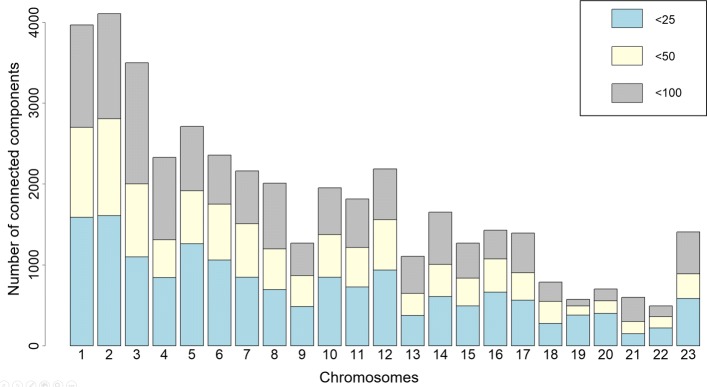
Connected component size distribution. The number of connected components with less than 25, 50 and 100 vertices for each chromosome. The components were computed using parameters *n*_*min*_=10,*n*_*max*_=100,*a*=0.75,*b*=0.25

**Fig. 6:**
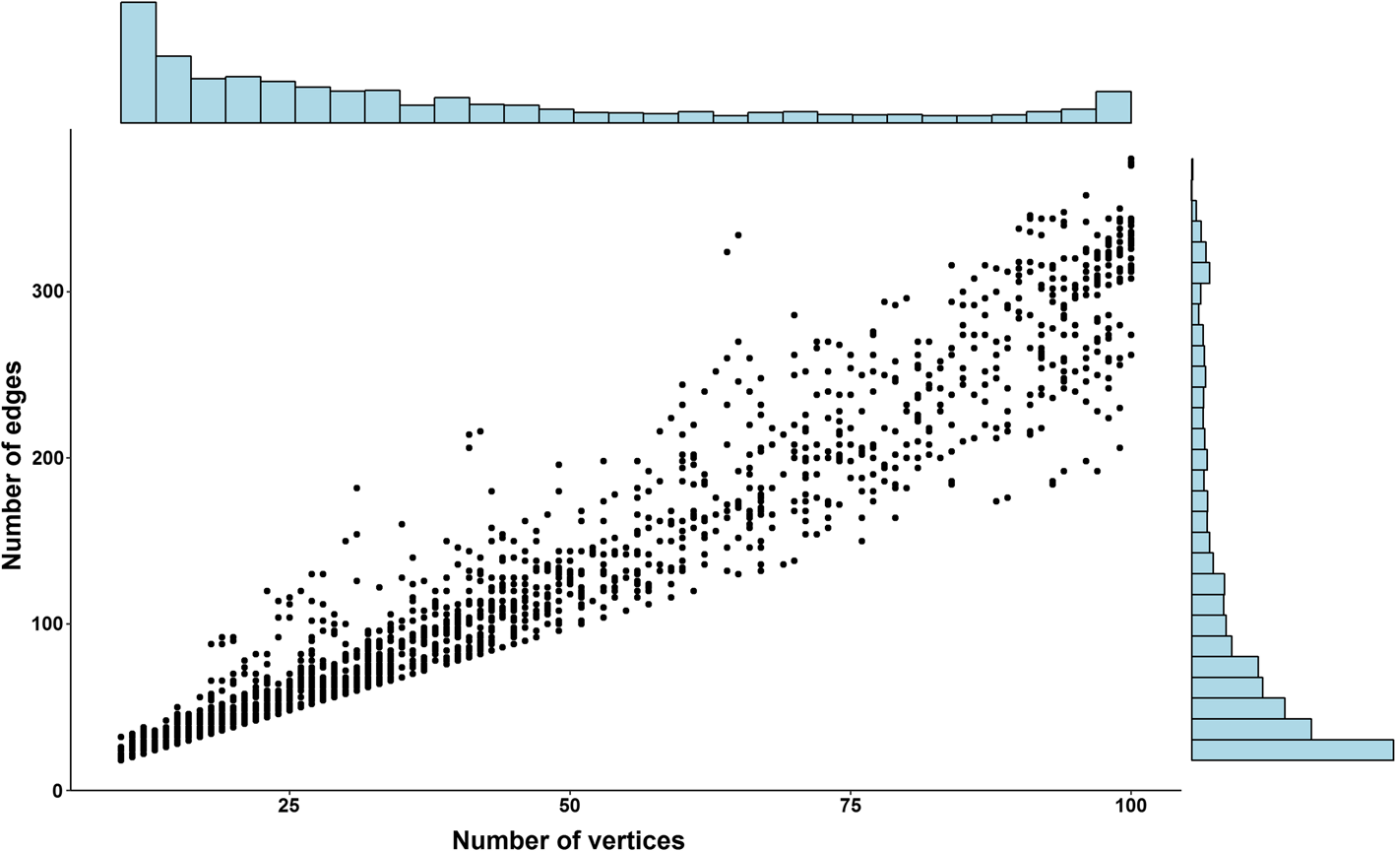
Vertex and edge numbers for connected components. Sizes of connected components in terms of a number of vertices and number of edges for each component. The particular size distribution is shown for chromosome 6, but distributions for other chromosomes are similar

The whole data set was analysed using the parameter values *n*_*min*_=10,*n*_*max*_=100,*a*=0.75 and *b*=0.25. The number of components found for each chromosome range between 492 and 4107 and is shown in Fig. 5 (additionally shown are the numbers of components with less than 25 and less than 50 vertices). The distribution of the component sizes (number of edges and number of vertices) is shown in Fig. 6 (the data are shown for chromosome 6 but distributions are very similar for other chromosomes). The average edge to vertex proportion within components is slightly larger than in the whole interaction network.

**Fig. 7:**
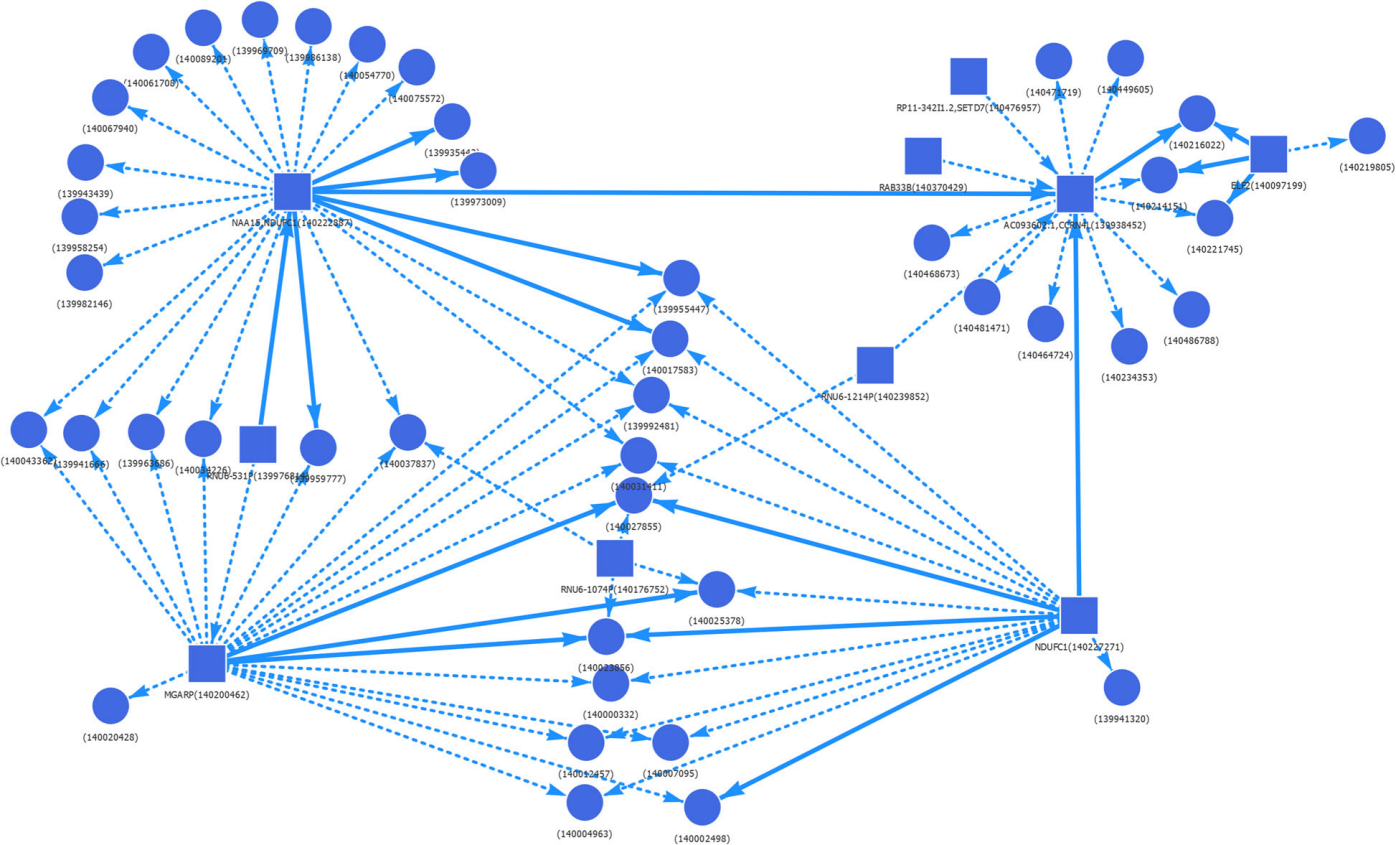
An example of a connected component with a comparatively high significance score. A connected component *C*≤_*cc*_*G*(*T*) of chromosome 4 with 53 vertices, 80 edges and significance score 6. All edges are labelled with all cell types from *T*={*nB,aCD*4,*Neu*}. For all *t*∈{*tB,tCD*8,*nCD*8,*naCD*4,*tCD*4,*nCD*4,*FoeT,Ery,Mon*} graphs *G*(*C,T*∪{*t*}) contain at least 75% of *C* edges and for all or all *t*∈{*EP,Mac*2,*Mac*1,*Mac*0} graphs *G*(*C,T*∪{*t*}) contain at most 25% of *C* edges. Un-dotted edges are the ones preserved in *G*(*C,T*∪{*EP*})

A ’typical’ larger component *C* with a comparatively high significance score 6 from chromosome 4 is shown in Fig. 7. It is defined by the set of labels *T*={*nB,aCD*4,*Neu*}. At least 75% of its 80 edges are preserved in subgraphs *G*(*C,T*∪{*t*}) with *t*∈{*tB,tCD*8,*nCD*8,*naCD*4,*tCD*4,*nCD*4,*FoeT,Ery,Mon*} and at most 25% edges are preserved in subgraphs *G*(*C,T*∪{*t*}) with *t*∈{*EP,Mac*2,*Mac*1,*Mac*0}. As an illustrative example the subgraph *G*(*C,T*∪{*EP*}) is swown.

### The structure of the ’component space’

From the number of components that are found by FINDNETWORKCOMPONENTS algorithm (Fig. 5) it is obvious that they must have overlapping sets of vertices. E.g. interaction graph for chromosome 1 contains 23079 vertices, however, the algorithm outputs 3066 components with at least 10 vertices each (the total number of vertices for all components is 162172).

By its design FINDNETWORKCOMPONENTS algorithm prunes the list of components found by not searching for any sub-components *C*^′^≤_*cc*_*G*(*C,T*^′^), if a component *C*≤_*cc*_*G*(*T*) has already been output as significant for some *T*⊆*T*^′^. This, however, does not exclude the possibility to output partially overlapping components, and from the results, it is clear that the amount of overlapping components is still significant.

The pruning also does not exclude finding components of *G*(*T*) and *G*(*T*^′^) with the same set of vertices if there is only a partial overlap of *T* and *T*^′^. The number of latter, however, is quite small.

**Fig. 8:**
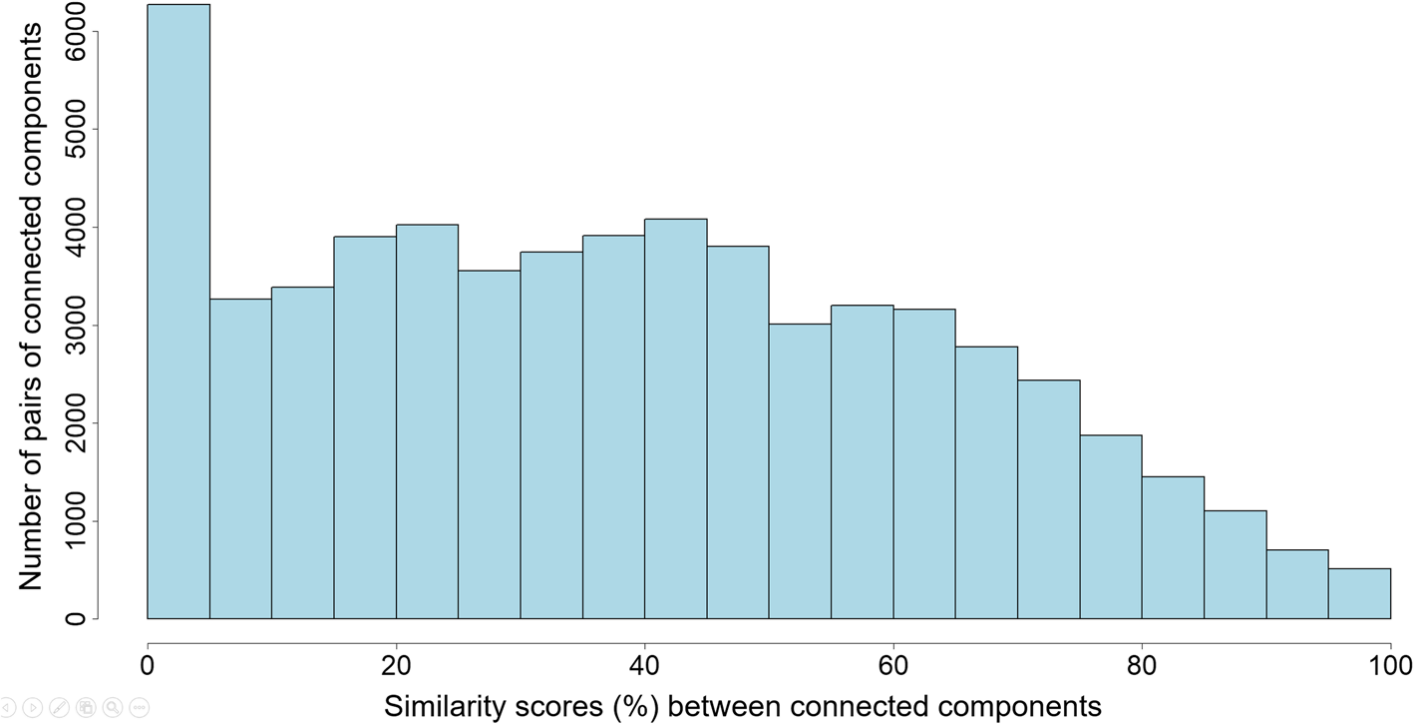
Distribution of similarity scores between connected components. Distribution of similarity scores (ranging from 0% to 100%) between connected components. Data are shown for chromosome 6, but distributions for other chromosomes are similar

To analyse the degree of overlapping between components we compared them using a simple similarity score. For two components *C* and *C*^′^ their similarity score is defined as |*V*(*C*)∩*V*(*C*^′^)|/|*V*(*C*)∪*V*(*C*^′^)| and ranges from 0 (for non-overlapping components) to 1 (for components with the identical set of vertices). For each of the chromosomes all-against-all comparison of components was performed and similarity scores between them have been computed. A sample distribution (for chromosome 6, however, the distributions for other chromosomes are similar) of similarity scores is shown in Fig. 8.

More informative probably is representation of component similarity by graphs (’CC graphs’) in which components correspond to vertices and edges connect pairs of components with similarity score above some given threshold. For analysis of such ’CC graphs’ it again is useful to look at their component structure. A ’good feature’ of these graphs is that they split into comparatively (i.e. close to complete graphs) components that remain mutually isolated. This can be considered as another indirect confirmation that component structure is characteristic to PCHi-C interaction networks and not the result of specific technique of network analysis. The number of components in ’CC graphs’ and their sizes depend on the similarity threshold used. Table 2 shows number of components of ’CC graphs’ for chromosome 6, their maximal and average sizes as well as density (edge density of components with *n* vertices and *m* edges is defined as 2*m*/*n*(*n*−1) and shows how close these components are to complete graphs) for similarity thresholds from 0.00 to 0.95. The average sizes and average densities are computed only for components with 4 or more vertices. The data for all 23 chromosomes and similarity threshold 0.75 are shown in Table 3.

**Table 2:** Dependence of a number of connected components, their maximal and average sizes, and edge density from similarity threshold used to construct ’CC graph’

*Similarity threshold*	*Number* *of* *components*	*Max size*	*Average size*	*Average density*
0.00	48	279	63.2	0.740
0.05	65	223	49.6	0.781
0.10	72	223	46.5	0.785
0.15	85	183	42.0	0.775
0.20	98	183	38.3	0.787
0.25	110	152	34.7	0.782
0.30	128	114	31.6	0.752
0.35	151	107	27.2	0.727
0.40	183	107	22.8	0.713
0.45	220	102	21.4	0.686
0.50	280	100	18.4	0.688
0.55	344	100	16.4	0.680
0.60	450	71	14.9	0.680
0.65	580	70	14.1	0.669
0.70	732	64	12.4	0.641
0.75	959	64	9.9	0.656
0.80	1212	63	8.8	0.653
0.85	1484	55	8.0	0.663
0.90	1789	41	6.7	0.706
0.95	2072	16	5.8	0.748

In ’CC graph’ components represented by vertices are connected by edges if their similarity exceeds the threshold value. The data are given for chromosome 6 but are similar for other chromosomes. The minimal component size is 1 even for similarity threshold 0. Edge density of components with *n* vertices and *m* edges is defined as 2*m*/*n*(*n*−1) and shows how close these components are to complete graphs. Average component sizes and average densities are computed taking into account only components with more 4 or more vertices

**Table 3:** Number of connected components, their maximal and average sizes, and edge density in ’CC graphs’ for all chromosomes

*Chromosome*	*Number of components*	*Max size*	*Average size*	*Average density*
1	1410	104	10.7	0.640
2	1540	120	10.8	0.690
3	1145	440	13.9	0.669
4	822	126	12.5	0.643
5	985	59	10.0	0.701
6	959	64	9.9	0.656
7	944	158	9.7	0.686
8	714	127	11.4	0.663
9	566	62	9.8	0.614
10	816	111	10.8	0.649
11	759	71	9.4	0.762
12	843	74	10.4	0.646
13	446	134	13.4	0.627
14	561	196	13.3	0.647
15	580	38	9.2	0.629
16	531	62	11.9	0.655
17	604	250	13.0	0.639
18	363	27	8.0	0.815
19	255	35	8.9	0.640
20	371	29	9.5	0.657
21	222	40	11.4	0.681
22	286	19	6.6	0.674
23	644	169	12.2	0.583

The similarity threshold used for constructing ’CC graphs’ is 0.75

**Fig. 9:**
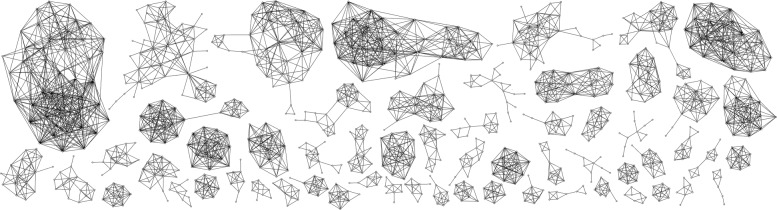
Relations between connected components with a similarity threshold above 75%. Graph showing similarity relations with a similarity score above 75%. Here components are represented by vertices and edges connect pairs of components with similarity score above 75%. Data are for chromosome 6 and only part of 2355 components are shown (the ones forming the largest connected parts of ’CC graph’, most components are contained in small connected fragments of 1 to 4 vertices, which are not shown here)

Part of ’CC graph’ for chromosome 6 is visualised in Fig. 9 (the largest part of the graph’s 2355 vertices, however, are contained within components of 1 to 4 vertices, which are not shown here). This figure shows a typical component structure of ’CC graphs’ regardless of the used similarity thresholds, although for the lower thresholds there are fewer components and average and maximal component sizes increase.

At present this description about the structure of ’component space’ is provided only as being potentially useful for interpretation of the results produced by FINDNETWORKCOMPONENTS algorithm. Nevertheless, it is also clear, that reducing the number of components by including only representatives from sets of very similar ones (and probably excluding some that are of little interest – e.g. quite typical ’star like’ structures) would facilitate analysis of their biological roles. However, this unlikely can be done on the basis of similarity scores alone. A second semi-supervised processing stage for selecting ’representative set’ from the components directly output by the algorithm remains an interesting option to explore.

### Assessment of the biological significance of network components

**Fig. 10:**
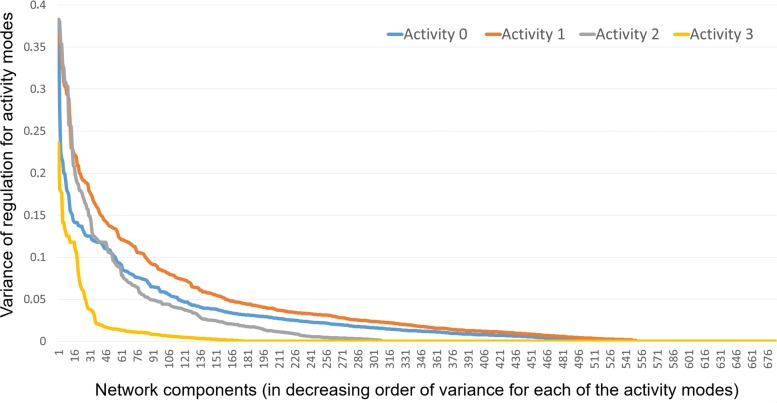
Variance of regulation activity modes within network components. Variance of regulation activity modes within network components (shown in decreasing order for each activity mode). For largest part of the components the variance values are below the overall variance (correspondingly 0.028, 0.040, 0.024 and 0.006 for activity modes 0, 1, 2 and 3). The data are shown for chromosome 6

**Fig. 11:**
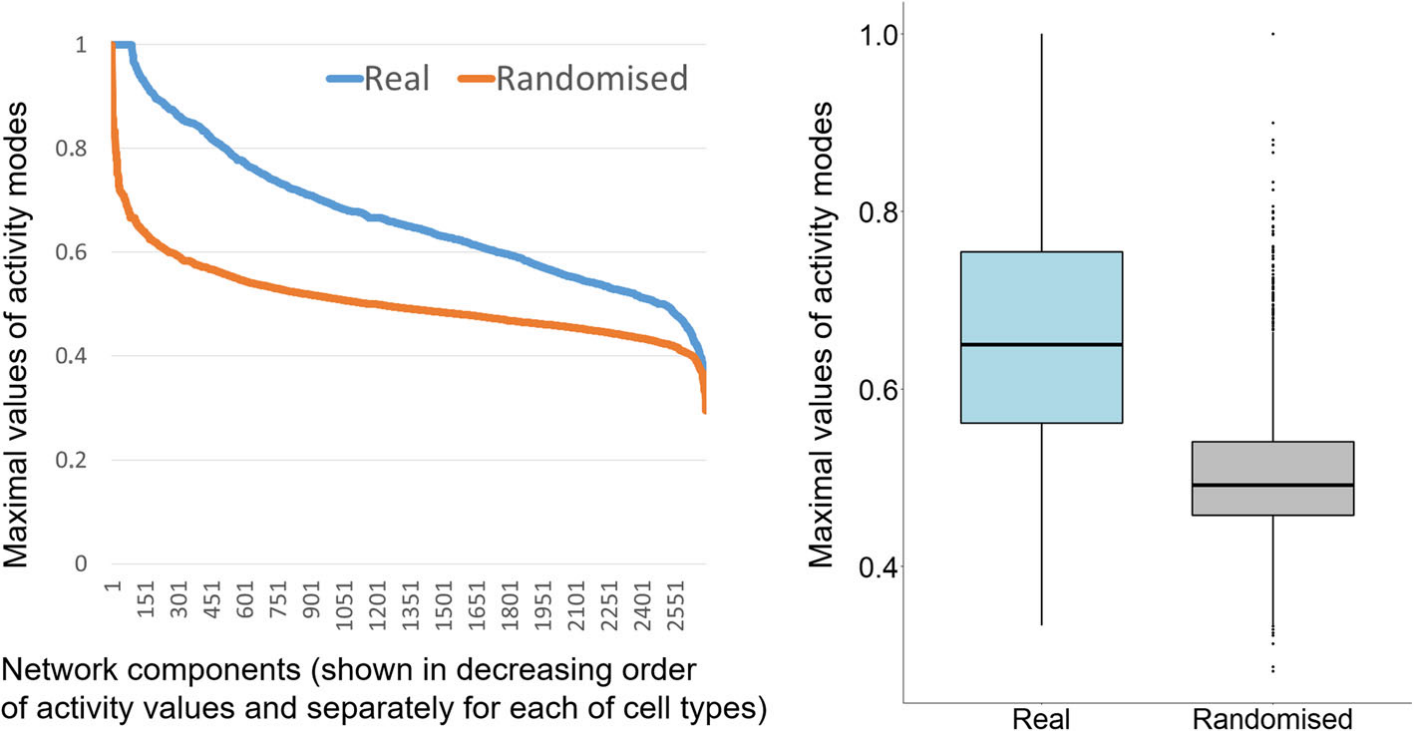
Distribution of maximal values of activity modes within connected components in comparison to randomised data. (Left) Distribution of maximal values of activity modes *a*_*i*_ within connected components (blue line) in comparison with value distribution when edges (with the same type labels) are assigned to components randomly (orange line). For both data sets the values are sorted in decreasing order with each point on *x* axis corresponding to one component limited to a single cell type. The activity values shown on *y* axis due to normalisation range between 0 and 1. The chart shows that edges within connected components tend to be significantly more associated to component-specific activity modes than it will be expected from random edge assignments to components. The *p*-value assigned by Wilcoxon test to statistical significance of this effect was 2.2×10^−16^ (i.e. ’almost zero’). (Right) Box plots showing distributions of real and randomised data. The data are shown for chromosome 6, but the same very low *p*-value was obtained for all the chromosomes

The assessment of the potential biological significance of the components was performed using gene regulation annotations, and the results show that *on average* the genes from the same component have similar regulation patterns, thus in principle confirming that components of PCHi-C interaction networks are related to specific biological functionality and/or biological mechanisms of their formation. A brief summary is given by Fig. 10 showing variance of regulation activity modes within network components, which for most of the components are below the overall variance values. There is also a strong tendency for each component being associated with this component-specific activity mode. This effect in comparison to the distribution for randomised data is shown in Fig. 11. The exact calculation of statistical significance of this effect is difficult due to complex dependencies between the component edges. We computed the approximate *p*-values with the Wilcoxon test [32] (using its standard implementation in R language; the test itself can be considered to be the most appropriate for the data that are not normally distributed). For all the chromosomes the obtained *p*-values were 2.2×10^−16^ (i.e. ’almost zero’) when comparing activity distributions between our components and randomised data. Whilst such estimate is not totally accurate, it is still a very convincing indication that the statistical significance is high.

The activity assessments provided for the predicted transcription start sites and enhancers in the whole PCHi-C dataset were essentially dominated by two major groups: active regions (activity "1") and dead zones (activity "0"), both making up approximately half of all activities observed. Poised (activity "2") and Polycomb-repressed (activity "3") sites were comparatively much rarer, together making up no more than 10−15*%* of all activities in every chromosome except for chromosome 17, where poised activity was predicted in 22% of sites in the whole data set. When compared to this overall background, our components showed mild but noticeable differences in the activity. Foremost of these was an enrichment in predicted dead zones, with a mean 5% increase across all chromosomes, the highest enrichment of which (13%) was observed in chromosome 13. Conversely, active sites seemed comparatively diminished in our components, showing a largely consistent decrease in prevalence across most chromosomes. Polycomb-repressed sites were mostly enriched as well but remained in the 1−3*%* range of prevalence.

When considering distributions of activities within components, we found that approximately two thirds of the components showed a strong primary activity predicted for 60% of sites or more. Much like in the overall distribution, most of these are dominated by either predicted active sites or dead zones. There are also approximately 100 components showing a majority of poised activity, but only three dominated by Polycomb repression. Moreover, almost all of these components show noticeably low differences in the activity types detected between the tissue types that they primarily belong to, with variances between tissue types generally well below those observed in random permutations of the same data (Fig. 10). Altogether these results point toward shared properties within individual components, although no unified significance that applies to every component can be isolated through our method.

## Discussion

Our initial goal in setting the criteria for suitable components was to find dynamic structural elements in chromatin corresponding to tissue-specific modules of promoter-enhancer interactions. Our results instead show that our components, and also the PCHi-C data they stem from, connect a wider variety of structures, including most notably a wide range of components that connect chromatin ’dead zones’ with an overall quiescent profile of histone marks [33, 34]. These components occur in roughly equal proportion to ones that are dominated by markers of activity, and in fact, are more frequent in our filtered component set compared to the full set of annotated edges. As such, an important question to ask would be how to differentiate such components in our data, if it is possible. Furthermore, it may be important to also consider other components previously filtered out in our selection process and to analyse what each of them may contain.

More elaboration on the content and selectivity of the components could potentially be obtained in several ways. Firstly, with further data from a wider variety of independent datasets such as the FANTOM5 promoter-level expression atlas [27]. Of particular note may be ATAC-seq and DNase accessibility studies, as DNA accessibility is an obligatory prerequisite of enhancer function, whereas particular histone marks do not necessarily enable or preclude such function in vivo [35]. Additionally, even with the data we have currently gathered, it may be possible to find further topological properties of components such as particular sizes or modes of connectivity which help differentiate between the components already found. As we have demonstrated in our previous work, metrics based on graph topology can be used to differentiate between broad categories of tissue types [21], and so may be useful in finding out the general properties of highly active and spatially compact gene clusters.

Our analysis could also benefit from the re-examination of the components we have filtered out in the present study. The current criteria stipulate that each component must be specific to a small number of tissue types, but still retain most of its interactions in at least one more tissue. Given that these criteria eliminated the majority of the connected components found in the original dataset, it is likely that we could glean some insight from both non-specific components found in most or all tissue types studied, and also highly specific components delineating the most variable elements of dynamic chromatin architecture. In fact, understanding the contribution of the latter category to the regulatory landscape of blood cells may be the logical next step in further work, with non-specific chromatin architecture serving as a useful frame of comparison.

Regarding the algorithm FINDNETWORKCOMPONENTS for identification of connected components of potential biological significance the part that likely can be further elaborated/improved is SIGNIFICANCESCORE function. In current version significance scores do not depend on the size of connected components and high scores are assigned also for comparatively small and simple components that (depending on the biological questions further asked) might not always be of particular interest. A better insight into component topology and its relation to biological functionality, however, is needed for this.

As already mentioned, reducing the number of components would facilitate analysis of their biological roles and a second semi-supervised processing stage for selecting ’representative set’ from the components directly output by the algorithm remains an interesting option to explore.

## Conclusions

In this paper we have presented a novel algorithm for analysis of chromatin interaction networks with a goal to identify characteristic topological features of interaction graphs and to ascertain their potential significance in chromatin architecture. The algorithm provides automatic identification of all connected components with significance score above the given threshold that can be potentially related to specific biological role or function. The fact that chromatin interaction networks tend to separate easily into well-defined connected components to which it is possible to assign certain biological functionality was previously observed by the authors [21]. However, identification of such components was only possible with manual ad-hoc methods, with which exploration of the whole component space was infeasible.

We have applied the developed FINDNETWORKCOMPONENTS algorithm to analysis of PCHi-C interaction networks of 17 different haematopoietic cell types based on dataset by [12]. This provided strong evidence that component structure for these PCHi-C interaction networks is quite pronounced and also allowed to obtain some characterisation of this component structure. We have also made assessment of potential biological significance of the found network components by analysing regulation patterns of genes contained within components and the results confirmed that *on average* genes from the same component have similar regulation patterns. At the same time it is very likely that the component structure is the result of number of different interrelated biological processes, and by no means we attempt to claim that we have assigned to them a unique well-defined biological explanation. What we think is significant, is the fact that component structure of PCHi-C interaction networks is sufficiently well manifested to be taken into consideration when analysing such networks.

The developed algorithm can be adapted for exploration of similar data sets of PCHi-C interactions (or chromatin interactions obtained by other technology) that includes information for sufficiently large number of different cell types. The analysis results obtained on another data set would provide significant new insights whether topological structure of the chromatin interaction networks is similar for different sets of cell types, and whether there are similar associations of structural components with specific biological functionality. Unfortunately, to our knowledge no other data set suitable for such a purpose as yet have become available, however, emergence of such data sets in near future is very likely. In particular, there are already available several genome-wide chromatin interaction data sets that cover few (up to 3) different cell types (e.g. [22]). Whilst data about only 3 cell types are really insufficient for component structure exploration (and the structure of ChIA-PET networks studied in [22] is somewhat different), the study provides an additional support to the hypothesis of importance of interaction network component structure. The authors of [22] have provided characterisation of biologically significant topological features of the networks in terms of graphlets (small subgraphs), although, in contrast to our approach, these have been derived from the known biological features, whilst our emphasis is on potential discovery of new biological relations from analysis of network topology.

More problematic, but still possible, is adaptation of the method for other types of cell (or tissue) type-specific networks. This could be quite feasible for gene regulatory networks (although instead of connected components, these likely will need to be analysed in terms of more complex topological structures); the main limitation, however, is the fact that currently the tissue-specific information on gene regulation is mostly provided by the chromatin interaction data. Still, a possible application could be analysis of gene regulatory networks of different species based on the known homologies between the genes. Although such task will be more complicated and it is difficult to predict how interesting and/or useful the results might be.

The developed software for automatic identification of components in PCHi-C interaction networks (C++ code and compiled Windows and Linux binaries) is publicly available at GitHub repository: https://github.com/IMCS-Bioinformatics/ PCHiCNetworkExplorer. The repository contains also data files describing analysed PCHi-C interaction networks used for experiments as well as analysis results – list of identified components with assigned scores of their anticipated biological significance. The software also includes a JavaScript based web-based browser for visualisation and exploration of components of PCHi-C interaction networks.

## Data Availability

The developed software for automatic identification of components in PCHi-C interaction network (C++ code and compiled Windows and Linux binaries) is publicly available on GPL license at GitHub repository: https://github.com/IMCS-Bioinformatics/PCHiCNetworkExplorer. The repository contains also data files describing analysed PCHi-C interaction networks used for experiments as well as analysis results – list of identified components with assigned scores of their anticipated biological significance. The software also includes a JavaScript based web browser for visualisation and exploration of components of PCHi-C interaction networks. This is a modified version of the browser that was previously made available as a supplement to an earlier publication of the authors [21].

## Abbreviations

3C: Chromatin conformation capture
aCD4: Activated total CD4+ T lymphocytes
ATAC-seq: Assay for transposase-accessible chromatin using sequencing
BFS: Breadth first search
CAGE: Cap analysis gene expression
CC: Connected component
CHi-C: Capture Hi-C
ChIA-PET: Chromatin interaction analysis by paired-end tag sequencing
CHiCAGO: Capture Hi-C analysis of genomic organization
ChIP: Chromatin immunoprecipitation
CTCF: CCCTC-binding factor
EP: Endothelial precursors
Ery: Erythroblasts
FoeT: Fetal thymus
Mac0: Macrophages M0 (non-activated macrophages)
Mac1: Macrophages M1 (inflammatory macrophages)
Mac2: Macrophages M2 (alternatively activated macrophages)
MK: Megakaryocytes
Mon: Monocytes
naCD4: Non-activated total CD4+ T lymphocytes
nB: Naive B lymphocytes
nCD4: Naive CD4+ T lymphocytes
nCD8: Naive CD8+ T lymphocytes
Neu: Neutrophils
PCHi-C: Promoter capture Hi-C
PEI: Promoter-enhancer interaction
tB: Total B lymphocytes
tCD4: Total CD4+ T lymphocytes
tCD8: Total CD8+ T lymphocytes
TSS: Transcription start site
